# Two Rare Cases of Non-Syndromic Paramolars with Family Occurrence and a Review of Literature

**DOI:** 10.3390/dj7020038

**Published:** 2019-04-01

**Authors:** Georgia Palikaraki, Emmanouel Vardas, Anastasia Mitsea

**Affiliations:** 1 Private Practice, 15772 Athens, Greece; gewpalhkarakh@gmail.com; 2 Department of Hospital Dentistry, Dental School, National and Kapodistrian University of Athens, Athens, Greece 2 Thivon str, 115 27, Goudi, 15780 Athens, Greece; emavard@dent.uoa.gr; 3 Department of Oral Diagnosis & Radiology, Dental School, National and Kapodistrian University of Athens, Greece 2 Thivon str, 115 27, Goudi, 15780 Athens, Greece

**Keywords:** paramolars, supernumerary teeth, non-syndromic, genetics, heredity

## Abstract

Supernumerary teeth (or hyperdontia) are teeth that exceed the normal number of deciduous or permanent teeth in the oral cavity. The occurrence of supernumerary teeth without any associated syndrome has been frequently reported and many case reports have been published. This article reports two rare cases of familial occurrence of multiple paramolars without the presence of any other syndrome for two consecutive generations. Limited cases of bilateral maxillary or mandibular paramolars have been reported. In addition, prevalence, classification, etiology, complications, diagnosis and therapeutic strategies that may be adopted when supernumeraries occur are discussed. A review of similar cases published in the literature is included as well.

## 1. Introduction

The most common dental anomaly in the deciduous and permanent dentition concerns numerical abnormalities. Supernumerary teeth (or hyperdontia) are teeth that exceed the normal number of deciduous or permanent teeth in the oral cavity [1].

Those teeth may be located anywhere in the oral cavity [2]. Although supernumerary teeth may occur bilaterally or even in multiples, they most commonly occur unilaterally. The prevalence of supernumerary teeth in permanent dentition ranges from 0.04% to 2.29%, while in deciduous dentition it ranges from 0.3% to 0.6% [3,4]. In permanent dentition, the frequency of supernumerary teeth is twice as common in males as it is in females [5].

Supernumeraries present a striking predilection for maxilla over mandible. The highest percentage (90%) of those is situated in the premaxillary region, especially in the central incisor region and in the midline. The other 10% is located in the mandibular premolar and maxillary canine regions, respectively [5]. More frequently, supernumerary teeth fail to erupt and remain impacted. Usually, supernumeraries are associated with several congenital genetic disorders or syndromes such as Gardner’s syndrome, cleidocranial dysostosis and cleft lip and palate. Other less common syndromes associated with supernumeraries are Fabry disease, Ellis–van Creveld syndrome, Nance–Horan syndrome, Rubinstein–Taybi syndrome and tricho-rhino-phalangeal syndrome [6,7].

The crown morphology of supernumerary teeth vary from normal to atypical while their roots may or may not be completely developed [8]. They are classified according to their location, form and shape. According to their location, they are classified into mesiodens, parapremolar, paramolar and distomolar. A mesiodens is situated in the midline maxillary area, while a parapremolar is situated between premolars and a paramolar is situated buccally or lingually to the molars or in the interproximal space between the second and third molars [9,10]. A distomolar is located distal to the third molar [11]. The forms of morphological variation are: conical—small peg shape (coniform), odontoma—supernumerary teeth with an irregular shape (tumor of odontogenic origin), supplemental—supernumeraries resembling adjacent unaffected teeth, tuberculate—multi-cusped and short barrel shaped teeth with a normal or invaginated crown but a rudimentary root [12]. According to their shape, they are categorized as supplemental or eumorphic (normal size and shape) and rudimentary or dysmorphic (abnormal shape and smaller size). Conical, tuberculate and molariform types are considered rudimentary supernumerary teeth [13]. The most common supernumerary teeth present conical morphology (44.5%), followed by tuberculate (38.7%) and supplementary (16.7%). However, other studies have obtained prevalence data that varies from 31–75% for conical, 12–28% for tuberculate, and 4–33% for supplementary teeth [14].

Unerupted and impacted supernumerary teeth are commonly revealed incidentally via a routine radiographic examination. The proper imaging technique is absolutely essential for a reliable evaluation of the prognosis and the appropriate therapeutic approach that must be separately adopted for each case [15].

This article reports two rare cases of familial occurrence of multiple paramolars without the presence of any other syndrome, for two consecutive generations. Limited cases of bilateral maxillary or mandibular paramolars have been reported. In addition, prevalence, classification, etiology, complications, diagnosis and therapeutic strategies that may be adopted when supernumeraries occur are discussed. A review of similar cases published in the literature is included as well.

## 2. Case Presentation

### 2.1. Case 1

#### Case 1 Presentation

**Figure 1:**
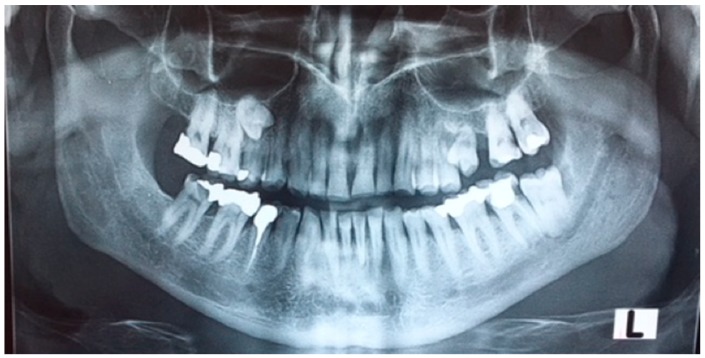
As seen in the panoramic radiograph of the father, the paramolars are located bilaterally in the maxilla. The right one is impacted, while the left one is partially erupted after the extraction of the first left molar.

**Figure 2:**
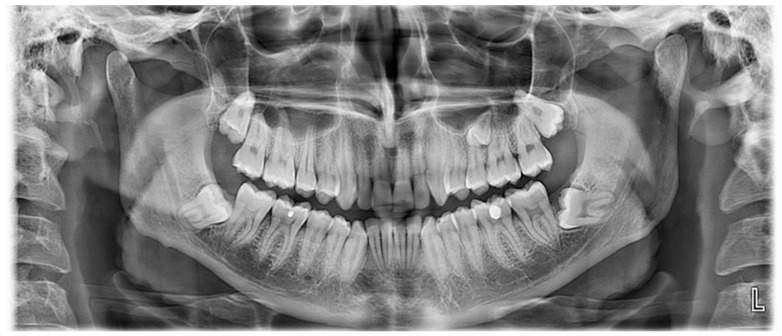
The four impacted third molars and the impacted paramolar are seen in the panoramic radiograph. The root of the paramolar seems to be close to the sinus maxillary wall.

A 65 year old male patient was referred to a private dental clinic with the chief complaint of pain in the lower second left molar. The patient’s medical history included hypertension and heavy smoking. His medical and dental history was noncontributory and there was no sign of any systemic diseases or syndromes. He mentioned that he had the upper left first molar extracted and the supernumerary premolar (that he already knew he had), erupted in this area. He also mentioned that his daughter had a supernumerary tooth in the same area but had had it extracted, hence her clinical records were not provided. A panoramic radiograph was performed on the patient, which incidentally revealed the existence of a supernumerary fully formed impacted paramolar located mesially to the upper right first molar (Figure 1). The crown of both paramolars (impacted and erupted) had two cusps and somewhat resembled a permanent premolar. The patient was informed of the existing condition and was advised to undergo a further radiological evaluation due to the potential risk of root resorption of the teeth adjacent to the impacted paramolars. Since both paramolars were asymptomatic, the patient was unwilling to proceed with further radiographic evaluation or removal of any of the teeth. Thus he was advised to undergo regular radiographic revaluation. After a two-month interval, the patient’s 25 year old son attended the same dental clinic, complaining of pain in the left area of the maxilla. Panoramic radiography examination revealed bilaterally impacted third molars in both jaws and a fully formed impacted paramolar. The root of the paramolar was positioned close to the left sinus maxillary (Figure 2). All of the radiographic findings and the potential risk of root resorption of the adjacent teeth was explained. The patient seemed unenthusiastic to proceed with further radiographic evaluation and intervention, so he was advised to undergo regular radiographic revaluation.

### 2.2. Case 2

#### Case 2 Presentation

**Figure 3:**
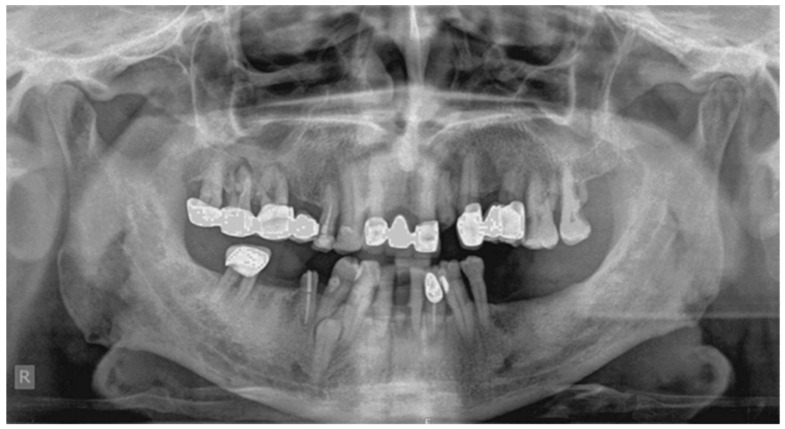
The parapremolar is located unilaterally, distally to the lower right canine.

**Figure 4:**
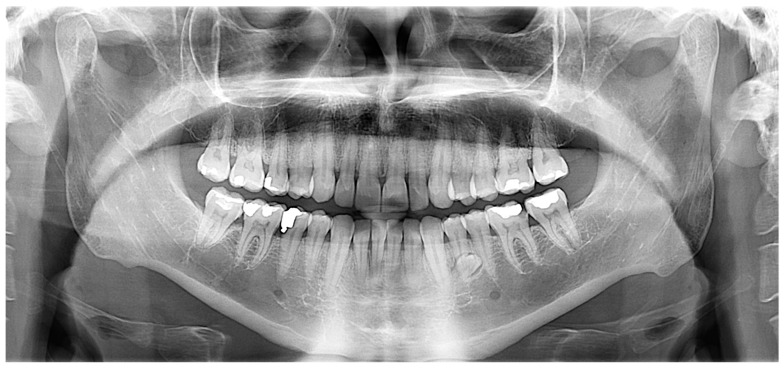
An incidental finding was the parapremolar on the left side of the mandible, located diagonally with its crown mesial to the second left premolar and its apex distal to the left second incisor.

**Figure 5:**
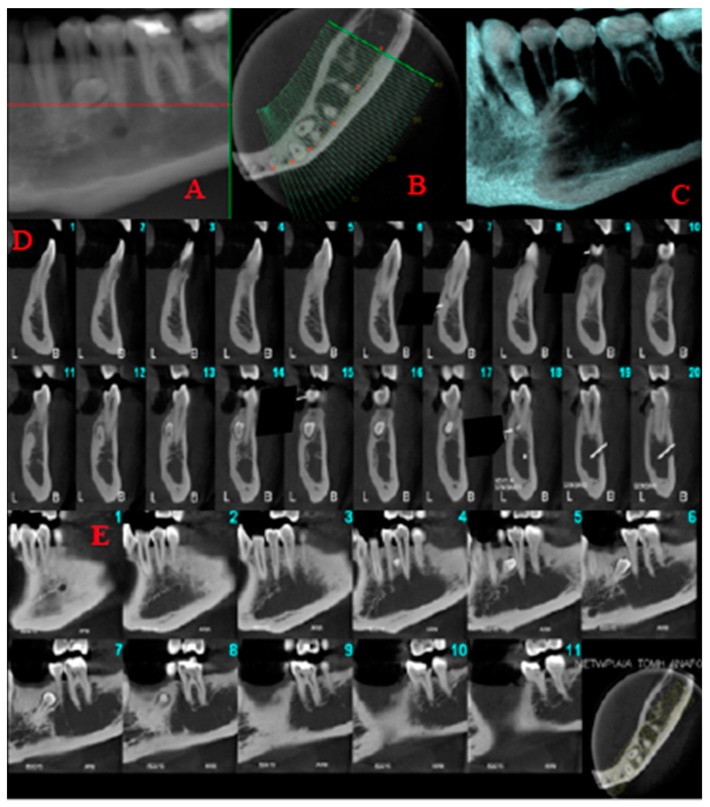
Limited field of view cone beam computed tomography images. Reformatted panoramic (**A**), axial (**B**), volumetric rendering (**C**) and sequential 1 mm thick/1 mm interval cross-sectional (**D**) and sagittal (**E**) cone beam computed tomography images of the left mandible demonstrating the supernumerary tooth, which is lingually located in the root of the lower left first premolar and is tangent to the latter.

A 60 year old male patient attended a private dental clinic with pain to the area of the upper left second molar. The patient’s medical history included hypertension, heavy smoking and alcohol consumption and he did not have syndromic features. His dental history included tooth mobility due to severe periodontitis. The panoramic radiograph incidentally revealed the existence of a fully formed impacted parapremolar located on the right side of the mandible between the first premolar and the canine (Figure 3). It was decided not to perform a cone beam computer tomography (CBCT) examination to evaluate the presence of root resorption of the adjacent teeth since they had a very poor prognosis. The patient’s 34 year old son attended the same dental clinic. In his medical history he reported frequent respiratory infections, the removal of his thyroid gland a year ago and he did not present any syndromic features. Concerning his dental history, his bilateral lower third molars had been extracted. An incidental finding in the panoramic radiograph of the son (Figure 4) was a fully formed parapremolar in the mandible, located diagonally with its crown mesial to the second left premolar and its apex distal to the left second incisor. In order to better evaluate the exact location of the parapremolar and the potential risk of root resorption to the adjacent teeth, a cone beam computer tomography (CBCT) was performed (Figure 5). As seen in the CBCT the parapremolar was located lingually. The supernumerary tooth as a whole was lingually located in the root of the lower left first premolar and was tangent to the latter. In particular, the distal portion of the supernumerary tooth’s crown was located proximal to the middle third of the root of lower left second premolar. The root of the supernumerary was located lingually and slightly below lower left canine. Moreover, it presented an abnormal morphology. The patient did not want to proceed with a surgical extraction of the supernumerary tooth and so observation was decided upon instead.

## 3. Discussion

The presence of supernumerary teeth is a common dental anomaly, but the occurrence of paramolars and parapremolars is relatively uncommon. A number of theories have been formulated to describe the etiology of supernumerary teeth; however, the etiology of this anomaly has not yet been clarified. The most commonly reported theories are the phylogenetic, the tooth germ dichotomy, the hyperactive dental lamina and the combination of genetic and environmental factors unified etiologic explanation [16]. As a result of phylogenetic evolution, the dimensions of the dental arches have been gradually redacted, followed by a decrease in teeth number and size. Thus, hyperdontia is related to a reversional phenomenon or atavism. Atavism is the tendency to return to primitive forms of features—in other words, the reappearance of features that had disappeared generations ago. Phylogenetic theory is not very strongly supported as it could only explain single anomalies of supernumerary teeth [5]. According to tooth germ dichotomy theory, the division of the tooth germ into two parts may occur as a result of an imbalance between molecules. These parts of equal or different size may form two teeth of the same size or one normal and one dysmorphic tooth, respectively [17]. According to the hyperactive dental lamina theory, the epithelial cells responsible for the formation of supernumerary teeth may persist for long periods [5]. Based on this theory the formation of supernumeraries is a consequence of localized, independent, conditioned hyperactivity of the dental lamina [13,18]. The hyperactive dental lamina theory is widely accepted; however, the most accredited theory is a combination of genetic and environmental factors unified etiologic explanation [18,19]. The aforementioned theory may be strengthened by the presence of supernumerary teeth in relatives of subjects with this dental anomaly [20]. Other studies that suggest genetic predisposition, as an etiological factor, are based on a dominant autosomal gene disorder [3,21]. However, the hereditary trait is not proved by a simple Mendelian pattern. A potential explanation may be low penetrance of dominant autosomal transmission. This is an implication that does not affect all generations [22]. In the aforementioned cases, familial occurrence of multiple paramolars or parapremolars without the presence of any other syndrome in two generations was presented.

The occurrence of supernumerary teeth without any associated syndrome has been frequently reported and many case reports have been published. However, a detailed literature review revealed very few reported cases of familial occurrence of non-syndromic supernumerary teeth (Table 1) [20,23,24,25,26,27,28,29,30,31,32,33].

Supernumerary teeth can be asymptomatic and are diagnosed as an incidental finding during radiographic examination [5,34]. On the other hand, the majority of unerupted teeth cause complications. These include dental impaction or ectopic eruption of an adjacent tooth, dilaceration or delayed or abnormal root development of associated permanent teeth or even the creation of follicular cysts from the degeneration of the follicular sac of the extra tooth. The most common complication is delayed eruption of the adjacent tooth, particularly with tuberculate morphology located palatally to the upper central incisors. A less common complication is overcrowding, mainly caused by supernumerary teeth in the anterior region of the maxilla. Frequently spacing anomalies, such as midline diastema, when the supernumerary is situated in the midline of maxilla, are also reported. Additionally, ectopic eruption of supernumeraries has been reported in several sites such as in the floor of the nasal cavity [35,36]. Complications may include malocclusion due to a reduction of space in the dental arch when the paramolar erupts and interdental spacing between molars is created. Dilaceration of the buccal mucosa due to traumatic bite when the paramolar is buccally positioned may also occur. Additionally, trigeminal neuralgia when the paramolar compresses the nerve, pulp necrosis and root resorption of the adjacent tooth have been reported [36]. Failure or ectopic eruption of a permanent tooth, persistence of a deciduous tooth and wide diastema may reveal the presence of supernumerary teeth [37]. In the presented cases, there were no complications.

Impacted supernumerary teeth are usually discovered incidentally during radiographic examination with no associated complications. Detailed dental history, clinical examination, early diagnosis and appropriate treatment of supernumerary teeth are mandatory. Clinical and radiographic evaluation of all teeth is crucial for the early detection of supernumerary teeth. There is no clear consensus on the appropriate time period for the removal of unerupted supernumerary teeth. Early intervention enhances therapeutic results [14]. By performing two-dimensional radiographic examinations such as anterior occlusal, a periapical radiograph using paralleling technique or a panoramic radiograph, basic useful information such as the existence, number and location of supernumerary teeth can be obtained. The panoramic radiograph seems to be the most effective two-dimensional imaging method for the initial evaluation of supernumerary teeth [1]. However, two-dimensional techniques present several limitations such as superimposition and weakness in presenting the three-dimensional relationship of adjacent anatomical structures and teeth. Moreover, it is impossible to evaluate if the tooth is located buccally or lingually and if it has caused any lesions to neighboring teeth. Cone beam computer tomography (CBCT) enables the clinician to accurately determine the exact location of each supernumerary tooth and to depict its orientation and sagittal position. Since CBCT provides significant information for pretreatment evaluation of supernumerary teeth, it is the recommended imaging technique in those cases [35].

The clinical management of patients with paramolars may differ from long-term follow-up to extraction, depending on multiple parameters. As long as the supernumerary tooth does not provoke any complication and does not interfere with function or aesthetics, long-term radiographic follow-up is the preferable approach. Moreover, systematic radiographic follow-up ensures the early diagnosis of new supernumerary teeth which are likely to occur in the future [37]. If it has been decided that the supernumerary should be removed, the difficulty of the procedure and the potential hazards to the adjacent anatomical structures during the intervention should be evaluated by performing the appropriate imaging technique.

In both presented cases, paramolars and parapremolars were an asymptomatic incidental finding. Since there was no evidence of any effect on the neighboring teeth or any other pathology, long-term radiographic follow-up was chosen.

To conclude, the formation of a supernumerary tooth indicates a genetic background of supernumerary teeth. The clinical implication is that if a supernumerary tooth is present, direct relatives of the patient should also be checked for the presence of supernumerary teeth. The latest progress in molecular biology research has provided us with significant information about tooth morphogenesis and differentiation. Mouse models are commonly used for studying tooth development and identification of candidate genes involved in the pathogenesis of supernumerary teeth. Few mouse models exhibit supernumerary teeth similar to those in humans. The current information, although informative, leaves many significant questions concerning the induction of supernumerary teeth [37]. Nevertheless, the initiation of tooth formation, the genetic control of successional teeth, as well as the mechanisms causing supernumerary tooth formation are still almost unclarified. Based on stem cell biology and tissue engineering, the exact etiology of supernumerary tooth formation might be clarified in the future. It is very important for a dental practitioner to be familiar with paramolars and parapremolars with regard to not only clinical complications, but also their management. Follow-up visits of those patients indicate that observational treatment can produce acceptable results.

## Figures and Tables

**Table 1:** Review of the literature [20,23,24,25,26,27,28,29,30,31,32,33].

Reference	Publication Year	Family Members Affected
Present case	2018	(1) Father, son and daughter (2) Father and son
Khambete and Kumar	2012	Father, son and two grandsons
Verma et al.	2010	(1) Two siblings (2) Father and son
Inhingolo et al.	2010	Three siblings
Orhan et al.	2006	(1) Mother and son (2) Mother and son
Batra et al.	2005	(1) Two siblings and father (2) Two siblings and mother
Cassia et al.	2004	Five members of a family
Shama	2003	Father and daughter
Umweni and Osunbor	2002	Two brothers and daughter
Galas and Garcia	2000	Two sisters
Marya and Kumar	1998	Two brothers
Seddon et al.	1997	Twins
Almeida et al.	1995	Three siblings
